# Intraspecific differentiation of *Lindera obtusiloba* as revealed by comparative plastomic and evolutionary analyses

**DOI:** 10.1002/ece3.11119

**Published:** 2024-03-11

**Authors:** Xiangyu Tian, Jia Guo, Yu Song, Qunfei Yu, Chao Liu, Zhixi Fu, Yuhua Shi, Yizhen Shao, Zhiliang Yuan

**Affiliations:** ^1^ College of Life Sciences Henan Agricultural University Zhengzhou Henan China; ^2^ School of Life Sciences Zhengzhou University Zhengzhou Henan China; ^3^ Key Laboratory of Ecology of Rare and Endangered Species and Environmental Protection (Ministry of Education) Guangxi Normal University Guilin Guangxi China; ^4^ Guangxi Key Laboratory of Landscape Resources Conservation and Sustainable Utilization in Lijiang River Basin Guangxi Normal University Guilin Guangxi China; ^5^ Center for Integrative Conservation, Xishuangbanna Tropical Botanical Garden Chinese Academy of Sciences Mengla Yunnan China; ^6^ College of Biological Resource and Food Engineering Qujing Normal University Qujing Yunnan China; ^7^ College of Life Sciences Sichuan Normal University Chengdu China

**Keywords:** complete chloroplast genomes, intraspecific diversity, *Lindera obtusiloba*, phylogenetic analysis, positive selection

## Abstract

*Lindera obtusiloba* Blume is the northernmost tree species in the family Lauraceae, and it is a key species in understanding the evolutionary history of this family. The species of *L. obtusiloba* in East Asia has diverged into the Northern and Southern populations, which are geographically separated by an arid belt. Though the morphological differences between populations have been observed and well documented, intraspecific variations at the plastomic level have not been systematically investigated to date. Here, ten chloroplast genomes of *L. obtusiloba* individuals were sequenced and analyzed along with three publicly available plastomes. Comparative plastomic analysis suggests that both the Northern and the Southern populations share similar overall structure, gene order, and GC content in their plastomes although the size of the plasome and the level of intraspecific variability do vary between the two populations. The Northern have relatively larger plastomes while the Southern population possesses higher intraspecific variability, which could be attributed to the complexity of the geological environments in the South. Phylogenomic analyses also support the split of the Northern and Southern clades among *L. obtusiloba* individuals. However, there is no obvious species boundary between var. *obtusiloba* and var. *heterophylla* in the Southern population, indicating that gene flow could still occur between these two varieties, and this could be used as a good example of reticulate evolution. It is also found that a few photosynthesis‐related genes are under positive selection, which is mainly related to the geological and environmental differences between the Northern and the Southern regions. Our results provide a reference for phylogenetic analysis within species and suggest that phylogenomic analyses with a sufficient number of nuclear and chloroplast genomic target loci from widely distributed individuals could provide a deeper understanding of the population evolution of the widespread species.

## Graphical abstract

*Lindera obtusiloba* in East Asia has diverged into the Northern and Southern populations, which are geographically separated by an arid belt. Here, ten chloroplast genomes of *L. obtusiloba* species were sequenced and analyzed along with three publicly available plastomes support the split of the Northern and the Southern clades and found a fewer photosynthesis‐related genes were under positive selection, which mainly related to the difference geological environments between Northern and Southern regions.

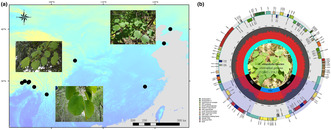

## 1 INTRODUCTION

Intraspecific variation is defined as genotypic and phenotypic diversity observed within a given species. Despite its significance in the taxonomic division, ecological processes, food security, and medicinal usage (Des Roches et al., 2018; Wilting et al., 2015), intraspecific variation has barely been taken into account when assessing biodiversity in current days (Díaz et al., 2018; Mimura et al., 2017; Ruckelshaus et al., 2020). Most evolutionary studies focus on interspecific variations (Alix et al., 2017; Aschehoug et al., 2016; Martín‐Serra & Benson, 2020) or genetic differentiation within a population (Halbritter et al., 2018; Taudt et al., 2016). Some efforts have been put into understanding the influence of biogeographic boundaries, environmental variables, and historical climatic instability on population genetics and phenotypic structure, especially intraspecific variations in species with a wide geographical distribution (Gray et al., 2019; Huang et al., 2016; Ye et al., 2017; Zhang et al., 2016). The demographic history of some species proves that subspecies living in separated subregions have experienced different selection pressures and have accumulated various mutations in their genomes (Tian et al., 2020). Intraspecific variation was recognized as a prerequisite for speciation (Bürger et al., 2006). Thus, a better description of the complexity of the genomic divergence among intraspecies, as well as the evolutionary processes, might help us identify species at risk of extinction and allow us to apply conservation strategies to protect them (Blomqvist et al., 2010; Leigh et al., 2019; Mimura et al., 2017).

*Lindera* Thunb. (spicebush or spicewood), one of the largest genera in the family Lauraceae, comprises almost 100 species, which are widely distributed in East Asia and East America (Chanderbali et al., 2001). *Lindera* is key to studying the origin and maintenance of tropical and subtropical plant communities, which are of significant economic and scientific value (Cao et al., 2016; Ye et al., 2017). Based on morphological characteristics (leaf venation, brachyblasts, pedunculate, fruit cupules, and so on), the genus *Lindera* has been divided into eight sections, namely sect. *Aperula*, sect. *Cupuliformes*, sect. *Daphnidium*, sect. *Lindera*, sect. *Palminerviae*, sect. *Polyadenia*, sect. *Sphaerocarpae*, and sect. *Uniumbellae* (Tsui, 1987). Due to the parallel evolution occurring between *Lindera* and *Litsea* Lam., proper classification of the two species or sections from the two genera proves to be difficult (Li, 1985). Previous studies have shown that nuclear loci (ITS, *rpb2*, etc.) and chloroplast regions (*matK*, *trnK*, *trnL‐trnF*, etc.) could not provide enough information to resolve the relationship between *Lindera* and *Litsea* and that species from the eight section of *Lindera* were placed in *Litsea* (Fijridiyanto & Murakami, 2009; Li, Conran, et al., 2008; Li, Li, & Li, 2008; Rohwer, 2000). Recently, some monophyletic clades, such as the sect. *Aperula*, sect. *Polyadenia* and trinerved *Lindera* complex have been successfully detected and classified via whole plastomic analyses (Fijridiyanto & Murakami, 2009; Li et al., 2004, 2007; Liu, Chen, et al., 2021; Liu, Ma, et al., 2021; Tian et al., 2019; Xiao et al., 2020). Although these detected monophyletic clades align well with morphology, the generic delimitation between *Lindera* and *Litsea* remains to be solved. Moreover, most phylogenetic studies have only sampled a single individual for each species, which is not sufficient for providing a consistent and reliable conclusion about the taxonomic status of that species (Liu, Chen, et al., 2021; Liu, Ma, et al., 2021; Xiao et al., 2022). It is also unclear whether the factors, such as hybridization or gene flow among intraspecific species, would drive taxonomic confusion among various varieties.

*Lindera obtusiloba* Blume is a deciduous shrub of the section *Palminerviae* in the genus *Lindera*, and it is widely distributed in the subtropical and temperate zones of East Asia. This species has diverged into var. *obtusiloba* and var. *heterophylla*, and this differentiation is based on the leaf margin morphology (Li, Conran, et al., 2008; Li, Li, & Li, 2008). The geological distribution of var. *heterophylla* is limited to the Southwest of East Asia while var. *obtusiloba* is widely distributed in East Asia. Despite of distributional difference, it is difficult to distinguish the two varieties based on morphology and molecular phylogeny in the Southern populations. It is of important economic value for it can be used as the raw material for the production of lubricants and fertilizers (Zekun & Haixia, 2012). Moreover, *L. obtusiloba* has also been used as a traditional medicinal herb for the treatment of liver damage and inflammation (Freise et al., 2010; Hong et al., 2013). *L. obtusiloba* is the Northernmost species in Lauraceae, and it is of great significance in studying the adaptation of Lauraceae plants to cold environments (Tian et al., 2020; Zhang et al., 2014). In previous studies, the divergence of *L. obtusiloba* into the Northern and Southern populations has been observed in studies with both chloroplast DNA and nuclear microsatellite markers (Ye et al., 2017). The two populations are geographically separated by an arid belt, which is modulated to be drier and wider during the Palaeogene (Guo et al., 2008). The arid belt acts as a barrier, which impedes gene flow between individuals living in the two sub‐regions (Bai et al., 2016; Chen & Lou, 2019; Ye et al., 2017), eventually leading to the formation of two separate populations (Tian et al., 2020; Xu et al., 2021). Previous studies have focused on population genetic diversity and the evolutionary history of this species. A divergence between the northern and southern populations of *L. obtusiloba* in East Asia has been observed in phylogenetic studies using cp DNA and SSRs. However, this divergence still lacks support from evidence at the genomic level.

The chloroplast is the organelle in which photosynthesis takes place, and it plays a vital role in energy conversion in plants (Ruhlman & Jansen, 2014). Chloroplast is a semi‐autonomous organelle, which contains its genome called plastome. Plastomes have often been used to investigate genetic differentiation and evolution of plant lineages. Plastome is smaller than mitochondrial and nuclear genomes in plant cells, and it has a large number of copies. The evolutionary rate of the chloroplast genome is higher than that of the mitochondrial genome but lower than that of the nuclear genome (Maple & Møller, 2006). The chloroplast genomes of land plants are relatively conserved in genomic structure, GC content, gene number, and gene arrangement (Xu et al., 2015). Structural variations have also been reported in diverse plant species, such as contraction and expansion in the IR regions, the loss of one IR, mixed presence of linear and circular genome, insertion or deletion, the loss of genes etc (Abdullah, Mehmood, et al., 2021; Abdullah, Shahzadi, et al., 2021; Alqahtani & Jansen, 2021; Lee et al., 2021; Li, Luo, et al., 2021; Li, Yang, et al., 2021; Sibbald & Archibald, 2020; Tonti‐Filippini et al., 2017). These characteristics of the chloroplast genome allow for its wide application in the research of evolutionary biology and molecular phylogeny, especially in the analysis of phylogenetic relationships at the intraspecific level.

In the current work, a total number of 10 newly sequenced *L. obtusiloba* chloroplast genomes with three published chloroplast genome sequences were compiled, which were classified into two populations. Geographically, the 13 samples allowed us (1) to evaluate the potential intraspecific variations in *L. obtusiloba*; (2) to assess whether the selection of the two varieties of *L. obtusiloba* is appropriate and sufficient for understanding the intraspecific relationship of this species, and (3) to reconstruct phylogeny between the Northern and Southern populations of *L. obtusiloba*. Based on the comparative chloroplast genome research, we expect to find considerable intraspecific divergence and extend our understanding of the complexity of the plastid divergence in widely distributed species.

## 2 MATERIALS AND METHODS

### 2.1 Sampling, DNA extraction, and chloroplast genome sequencing

**FIGURE 1:**
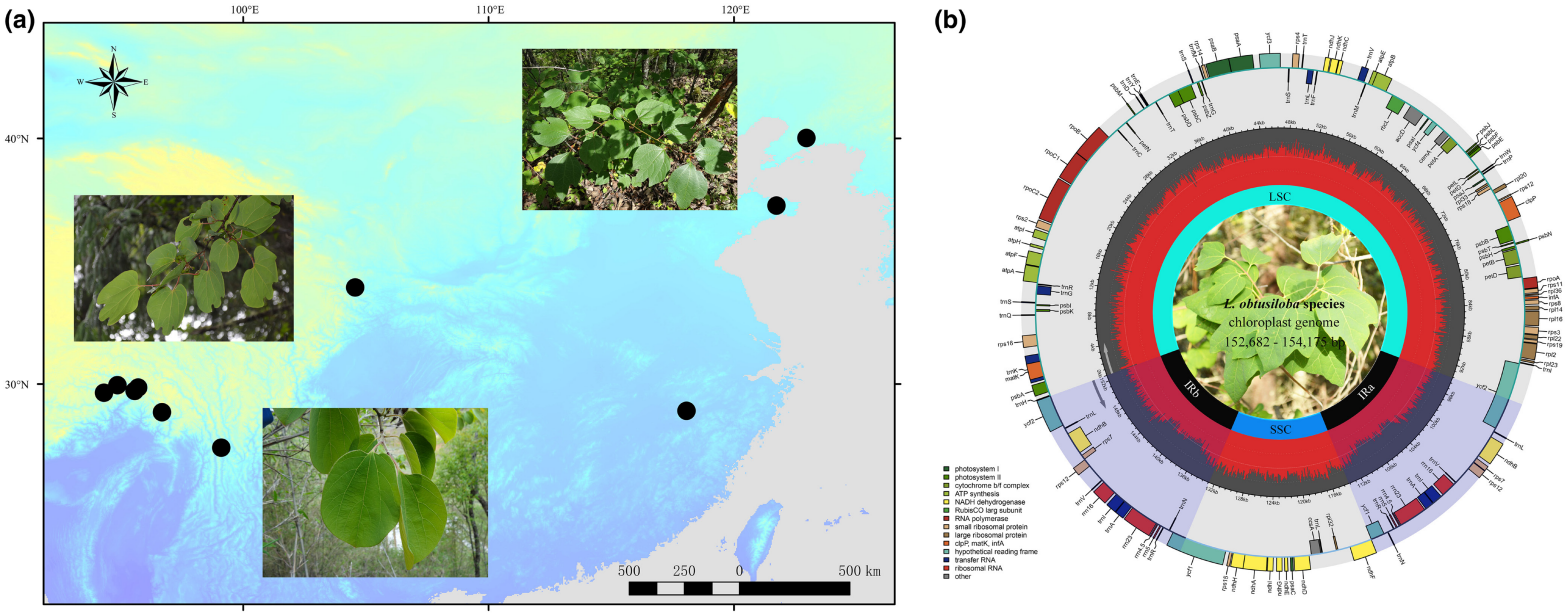
Samples location of the *Lindera obtusiloba* (a) and their gene maps of the complete chloroplast genome (b).

Fresh leaves from 10 individual trees of *L. obtusiloba* var. *obtusiloba* representing local populations were collected and silica‐gel dried (Table 1). The 10 samples include two individuals collected from Northeast China (the Northern population) and eight from Southeast to Southwest China (the Southern population) (Figure 1a). Voucher specimens were deposited in the herbarium of the School of Life Sciences, Henan Agricultural University, and the Herbarium of the Xishuangbanna Tropical Botanical Garden (HITBC), Chinese Academy of Sciences. Total genomic DNA was isolated from dry leaves using the Tiangen Plant Genomic DNA Kit (Tiangen Inc., China). DNA purity was examined using Qubit 2.0 (Invitrogen Inc., USA) and NanoDrop (Thermo Scientific Inc., USA). Purified total DNA was used to make DNA libraries using the Illumina Paired‐End DNA Library Kit and sequenced with the NovaSeq 6000 platform, a service provided by NovoGene Bio‐Tech (Beijing, China). Raw reads were obtained with an average length of 150 bp, yielding at least 4 GB of clean data for each sample. The sequence data of three formerly published chloroplast genomes of *L. obtusiloba* were retrieved from the NCBI database and included in this analysis, with two individuals belonging to the Northern population from Liaoning, China (GenBank No. MH220737) (Zhao et al., 2018) and Korea (GenBank No. MG581448) (Jo et al., 2019), respectively, and one individual of var. *heterophylla* from Tibet (GenBank No. MT621623) (Li, Luo, et al., 2021; Li, Yang, et al., 2021) representing the Southern population.

**TABLE 1:** Features of the ten *Lindera obtusiloba* new sequenced plastomes in the present study.

Distribution		Specimen number	GenBank no.	Genome size (bp)	Large single copy (LSC; bp)	Inverted repeats (IR; bp)	Small single copy (SSC; bp)	GC content (LSC/IR/SSC)	Number of genes (CDS/tRNA/rRNA)
Northern population	Yantai, Shandong	txy18016	ON520653	154,175	93,751	20,762	18,900	39.14% (37.92%/44.29%/33.80%)	113 (79/30/4)
	Dalian, Liaoning	sy8679	ON520651	152,770	93,700	20,080	18,910	39.12% (37.91%/44.44%/33.80%)	113 (79/30/4)
Southern population	Shangrao, Jiangxi	txy18043	ON520654	152,683	93,633	20,072	18,906	39.17% (37.97%/44.44%/33.88%)	113 (79/30/4)
	Longnan, Gansu	sy9339	ON520652	152,343	93,313	20,066	18,898	39.22% (38.06%/44.46%/33.85%)	113 (79/30/4)
	Diqing, Yunnan	sy5749	ON520645	152,682	93,630	20,071	18,910	39.16% (37.97%/44.45%/33.84%)	113 (79/30/4)
	Linzhi, Tibet	sy6219	ON520649	152,708	93,651	20,071	18,915	39.14% (37.95%/44.45%/33.80%)	113 (79/30/4)
	Linzhi, Tibet	sy7768	ON520650	152,708	93,651	20,071	18,915	39.14% (37.95%/44.45%/33.80%)	113 (79/30/4)
	Linzhi, Tibet	sy5779	ON520646	152,708	93,651	20,071	18,915	39.14% (37.95%/44.45%/33.80%)	113 (79/30/4)
	Chayu, Tibet	sy5998	ON520647	152,708	93,636	20,075	18,922	39.15% (37.96%/44.44%/33.82%)	113 (79/30/4)
	Chayu, Tibet	sy6045	ON520648	152,708	93,636	20,075	18,922	39.15% (37.96%/44.44%/33.82%)	113 (79/30/4)

### 2.2 Chloroplast genome assembly and annotation

The 10 complete chloroplast genomes were de novo assembled using the GetOrganelle toolkit with a typical setting (Jin et al., 2020) with the published plastomic data of *Lindera* from the NCBI database as a seed file. Annotation of the newly sequenced chloroplast genomes was performed with Plastid Genome Annotator (PGA) using the published *L. obtusiloba* (Accession no. MG581448) and *L. obtusiloba* var. *heterophylla* (Accession no. MT621623) plastome as a reference (Qu et al., 2019). To ensure the accuracy of the annotation, the same procedure was repeated with the GeSeq program (Tillich et al., 2017), which contains the HMMER profile and the tRNAscan‐SE server. The GB2sequin program was used to prepare the NCBI submission file (Lehwark & Greiner, 2019). Both programs were implemented in the CHLOROBOX web toolbox (https://chlorobox.mpimp‐golm.mpg.de/index.html). The online program of Chloroplot was used to visualize the circular genome physical map of *L. obtusiloba* (Zheng et al., 2020).

### 2.3 Whole chloroplast genome comparison

Comparative analyses across the 13 chloroplast genomes of *L. obtusiloba* were performed. The MISA software was used to identify simple sequence repeats (SSRs) with the following criteria: 10, 5, 4, 3, 3, and 3 repeat units are for mono‐, di‐, tri‐, tetra‐, penta‐, and hexa‐nucleotides, respectively (Beier et al., 2017). The REPuter web program was used to detect the forward, palindrome, reverse, and complement repeated elements with a minimal length of 30 bp, an identity value of more than 90%, and a Hamming distance of 3 (Kurtz et al., 2001). The intra‐genetic variations were analyzed and plotted using the mVISTA online tools with the Shuffle‐LAGAN mode (Frazer et al., 2004). To evaluate the significance of the analyses, three datasets representing the total population, the Northern population, and the Southern population were selected and analyzed. Nucleotide diversity (Pi) for each dataset was detected using the DnaSP software (Rozas et al., 2017). Furthermore, the sliding window analysis was conducted with a reference nucleotide diversity of 0.008 with a step size of 200 bp and a window length of 400 bp.

### 2.4 Phylogenomic analyses

The phylogenetic relationship of individuals within *L. obtusiloba* and to some related species from the “core” Laureae was interpreted using two separate datasets, the whole chloroplast genome and the protein‐coding genes (PCGs). A total of 65 chloroplast genomes representing 54 species or subspecies were included in this analysis, which covers the seven genera of the “core” Laureae. The species *Cinnamomum camphora* (L.) Presl belonging to the genus *Cinnamomum* Schaeff was used as the outgroup (Appendix S1). A multiple sequence alignment was generated with MAFFT under a default setting (Katoh & Standley, 2013) and then manually adjusted in BioEdit (Hall et al., 2011). Phylogenetic trees were constructed using both Maximum likelihood (ML) and Bayesian inference (BI) methods. The best‐fit nucleotide substitution model is GTR + F + R9 for the whole plastomic sequences and GTR + F + R3 for the coding regions determined with ModelFinder using the AIC values as criteria (Kalyaanamoorthy et al., 2017). In the ML analysis, IQ‐TREE (Nguyen et al., 2014) was implemented, and a bootstrap value of 50,000 was assigned with the SH‐aLRT branch test. In the BI analysis, MrBayes (Ronquist et al., 2012) was employed with two independent Markov Chain Monte Carlo chains for 2,000,000 generations. The first 25% of trees were discarded, and the split frequency is below 0.01.

### 2.5 Selective pressure analysis

Forty‐five protein‐coding genes were used to search for signs of positive selection in the *L. obtusiloba* chloroplast genomes with EasyCodeML (Gao et al., 2019). The unrooted phylogenetic tree file of *L. obtusiloba* species was generated using the ML approach. The ratio (ω) of the nonsynonymous substitution rate (dN) to the synonymous substitutions rate (dS) for each protein‐coding gene was determined using both the site model and the branch model. Signatures of adaptation were analyzed using the site model with four site‐specific models (M0 vs. M3, M1a vs. M2a, M7 vs. M8, and M8a vs. M8). The branch model was adopted under the one‐ratio model (M0) and the two‐ratio model with the Northern population of *L. obtusiloba* as the foreground branch. In addition, a likelihood ratio test (LRT) was performed to assess the significance of these analyses (Yang, 1998).

## 3 RESULTS

### 3.1 General characteristics of *L. obtusiloba* chloroplast genomes

The 13 *L. obtusiloba* plastomes share a conserved quadripartite circular structure with an overall length ranging from 152,682 bp to 154,175 bp (Figure 1b). The plastome size of the Northern population is comparably larger than that of the Southern population (Table 1). These chloroplast genomes all contain a large single‐copy region (LSC; length: 93,313 bp to 93,751 bp) and a small single‐copy region (SSC; length: 18,898 bp to 18,922 bp), which are separated by two inverted repeat regions (IRa and IRb; length: 20,066 bp to 20,762 bp) (Table 1). All individuals from the North possess a larger LSC region when compared to the Southern population. Species from Liaoning (a city in North China) share a similar IR size with individuals from the Southern population. The individuals from Gansu, which belongs to the Southern population, have the smallest SSC region. All individuals from Tibet share similar genome sizes, LSCs, IRs, and SSCs. In addition, the GC contents of all sampled plastomes vary slightly between 39.12% to 39.22%. Moreover, the GC content of IRs (44.29–44.45%) was higher than that of LSC (38.06–37.96%) and SSC (33.80–33.88%) regions. All the analyzed chloroplast genomes harbor 113 unique genes, including 79 PCGs, 30 tRNAs, and 4 rRNAs. Five PCGs and 6 tRNA genes are duplicated in the IR regions. The *rps12* gene was identified as a trans‐spliced gene with two duplicates in the IR regions and one in the LSC region.

### 3.2 Comparative phylogenomic analyses of *L. obtusiloba* plastomes

**FIGURE 2:**
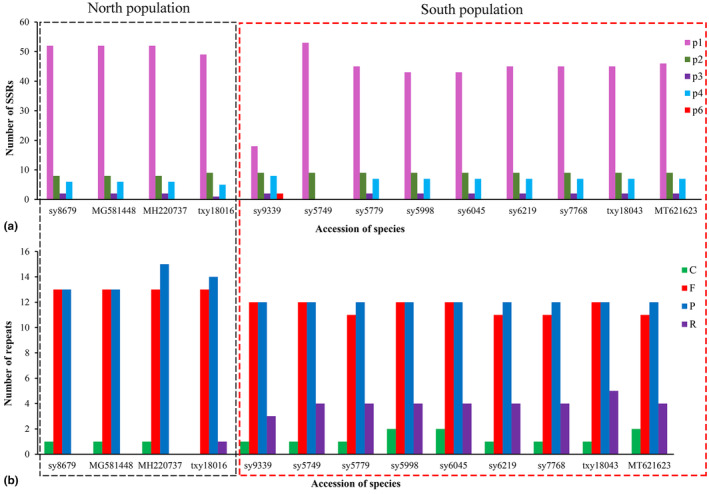
Number of repeats in the thirteen *Lindera obtusiloba* chloroplast genomes. (a) Total number of SSRs detected in each species. (b) Total number of the forward (F), palindrome (P), reverse (R), and complement (C) repeated elements detected in each species.

In this study, comparative analyses of the 13 plastomes were performed. A total of 371 longer dispersed repeats were detected and classified into 4 categories including forward, reverse, complement, and palindromic direct match repeats (Appendix S2). All individuals have a similar number of longer dispersed repeats, ranging from 20 to 27. The number of forward and palindromic repeats is 11–13 and 12–15, respectively. Among all the analyzed plastomes, the Northern *L. obtusiloba* individuals do not have reverse repeats except for the sample from Shandong, which has one reverse repeat but no complement repeats (Figure 2a). A total of 807 SSRs were identified among all species, which fall into 5 classes: mono‐, di‐, tri‐, tetra‐, and hexa‐nucleotide repeats (Appendix S2). All of the Northern individuals contain a larger number of mono‐nucleotide repeats than that of the Southern population (Figure 2b). The number of mono‐, di‐, tri‐, and tetra‐nucleotides is 52, 8, 2, and 6, respectively, for the Northern population, while it is 49, 9, 1, and 6 for spices from Shandong (Appendix S2). Among the Southern population, individuals from Gansu contain all five classes of repeats, while the number of mono‐nucleotide repeats (18) is relatively low. It is notable that the individuals from Yunnan only have mono‐ and di‐nucleotide repeats. Among the SSRs, the mono‐nucleotide repeats are the most frequent type with a typical A/T repeat unit, followed by the di‐nucleotide repeats, which are composed of TA/AT and/or GA/TC. The most commonly observed tetra‐nucleotide repeat units are AAAT/TTTA, AACT, AATG/CATT, ATAC, CATA, TTAT, and TTTC. Tri‐ (AAT and TAT) and hexa‐nucleotide (TAGAAT and TTCTAT) repeats are relatively rare (Appendix S2).

**FIGURE 3:**
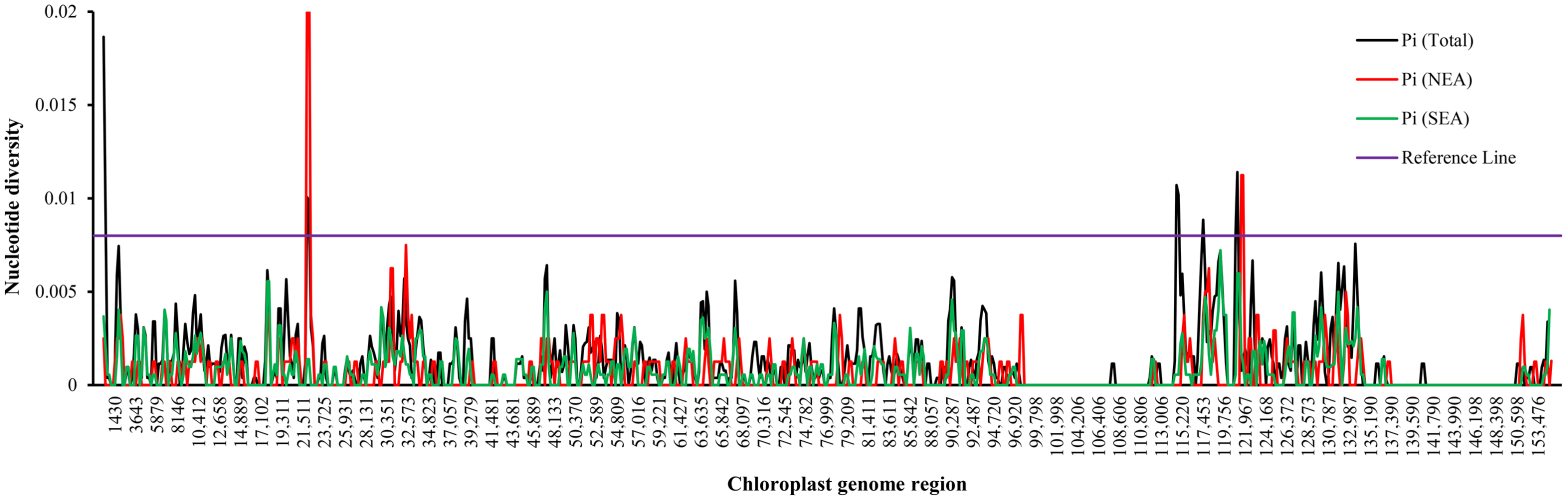
Sliding window analysis of the entire chloroplast genome of thirteen *Lindera obtusiloba* individuals (window length: 600 bp; step size: 200 bp). *x*‐axis: position of the midpoint of a window; *y*‐axis: nucleotide diversity of each window.

**FIGURE 4:**
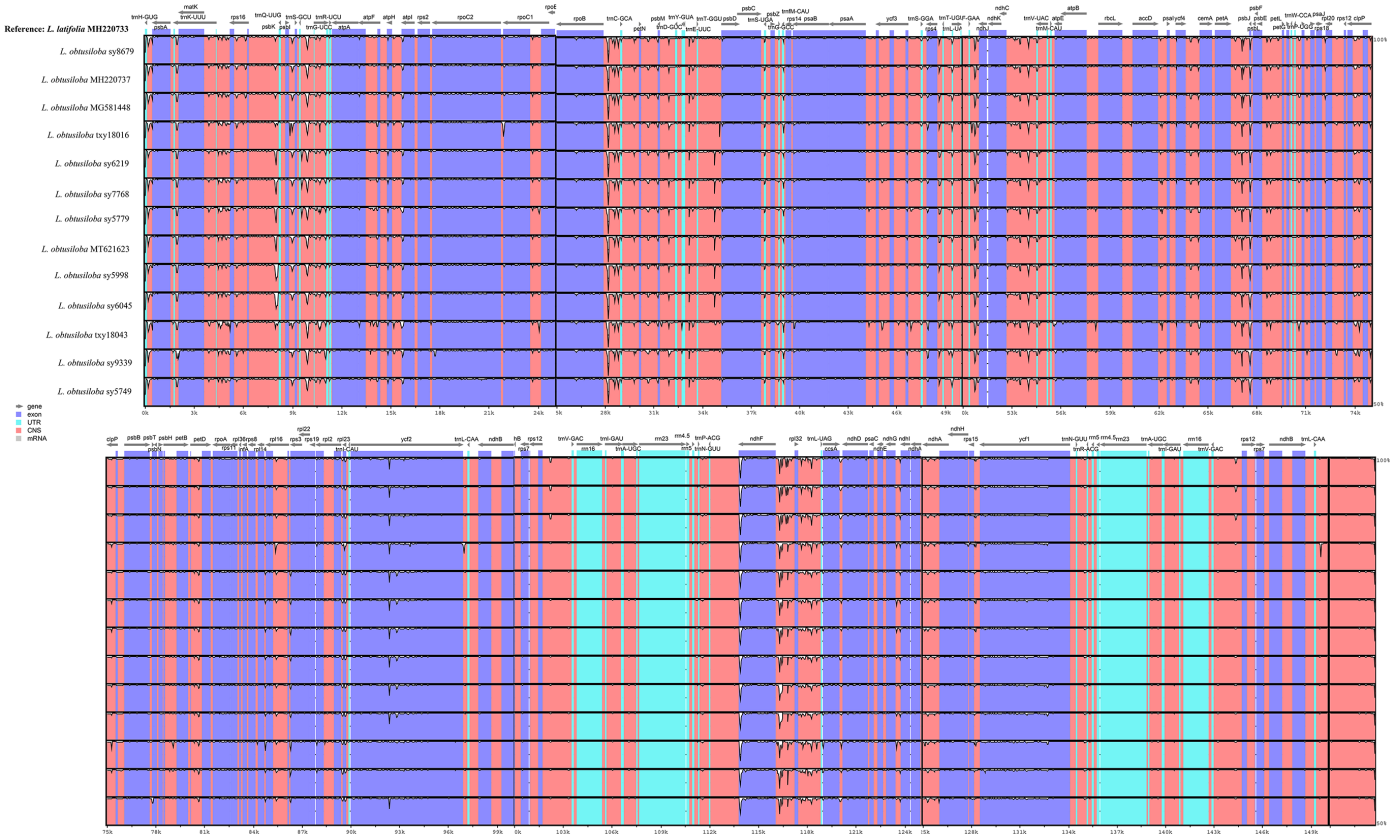
Visualization of genome alignment of the thirteen complete chloroplast genomes from *Lindera obtusiloba*. The cp genome of *Lavandula latifolia* is used as the reference. *x*‐axis indicates the sequence coordinates in the whole cp genome. *y*‐axis represents the similarity of the aligned regions, indicating percent identity to the reference genome (50–100%).

Nucleotide variations among intra‐ and inter‐populations of *L. obtusiloba* were also investigated in this work. The comparison suggests a high level of divergence among populations but a low level of divergence within a given population of *L. obtusiloba* (Table 2). A total of 329 singleton variable sites, 371 parsimony informative sites, and 340 InDels were identified across the whole chloroplast genomes with a nucleotide diversity value (π) of 0.00136. The coding regions are more conserved than the noncoding regions, with a π of 0.00118, which includes 141 singleton variable sites, 167 parsimony informative sites, and 71 InDels. Nucleotide diversity level was highly similar between the Northern (π = 0.00075 and 0.00066) and the Southern (π = 0.00076 and 0.00065) populations across the whole chloroplast genomes and the PCGs. However, the proportion of parsimony informative sites and InDels in the Southern population is higher than that of the Northern population (Table 2). Hypervariable regions of all *L. obtusiloba* were identified by the slide window test (Figure 3). Four regions possessing a nucleotide diversity larger than 0.008 were found, which are *trnH* ^‐GUG^‐*psbA*, *rpoC2*‐*rpoC1*, *ycf1*‐*ndhF*, and *ccsA*‐*ndhD*. In the Northern individuals, *rpoC2*‐*rpoC1* and *ccsA*‐*ndhD* show higher nucleotide diversity, while variable regions were not observed in the Southern individuals. With the mVISTA program, the sequence divergence of *L. obtusiloba* among all examined individuals was detected and analyzed with the *Lavandula latifolia* Hook. f. sequence as reference (Figure 4), the whole chloroplast genomes are conserved in structure and gene order. The non‐coding regions are more likely to accumulate variations than the coding regions, and IR regions are more conserved than the LSC and SSC regions.

**TABLE 2:** Comparison of the variable sites of the 13 *Lindera obtusiloba* plastomes.

Sequences	Variables	North population	South population	Total
Whole genome sequence	Singleton variable sites	229	238	329
	Parsimony informative sites	1	142	371
	Nucleotide diversity	0.00075	0.00076	0.00136
	InDels events	99	215	340
Protein coding sequence	Singleton variable sites	102	108	141
	Parsimony informative sites	1	59	167
	Nucleotide diversity	0.00066	0.00065	0.00118
	InDels events	24	43	71

### 3.3 Phylogenetic analysis of *L. obtusiloba* within the “core Laureae”

To gain more insights into the relationship among various *L. obtusiloba* individuals and other closely related species, a total of 65 chloroplast genomes including 13 *L. obtusiloba* individuals and other 51 “core Laureae” species were used for further phylogenetic analysis, which was conducted with both ML and BI methods using both the complete chloroplast genomes and the shared protein‐coding genes.

**FIGURE 5:**
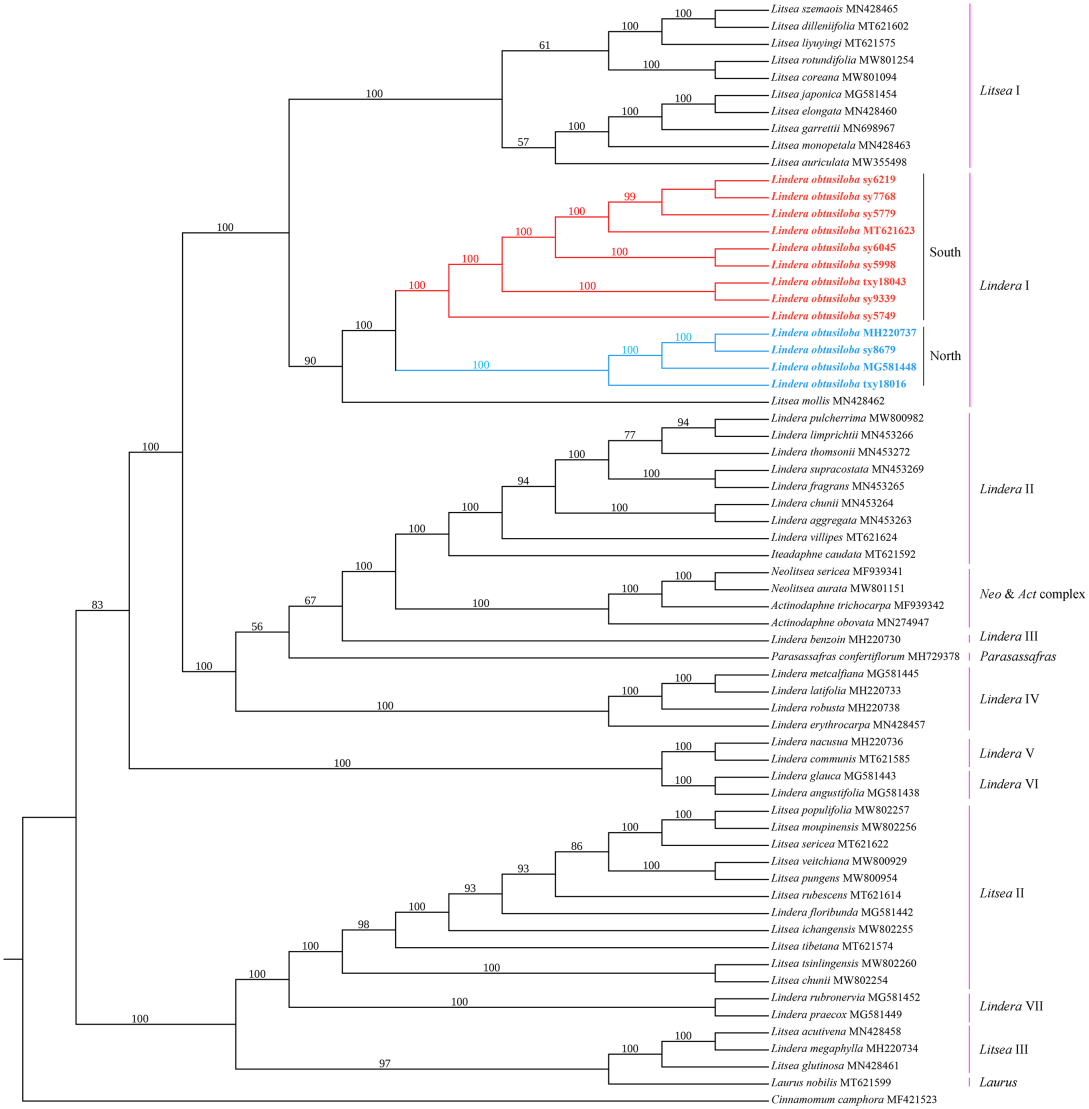
Phylogenetic tree reconstruction of ‘core’ Laureae using the maximum likelihood (ML) method based on complete chloroplast genome sequences. Only the ML tree is shown, because its topology is identical to that of the obtained BI tree.

Independent of the data and the inference method used, the obtained trees share a similar topology except for the *Lindera* IV‐VI clades, which contain the *Lindera* sect. *Aperula*, sect. *Polyadenia*, sect. *Lindera*, respectively (Figure 5). In the plastome‐based phylogeny, the *Lindera* IV clade represents a sister lineage of a larger group containing the genus *Parasassafras*, the North America *Lindera* species (*Lindera* III), genus *Neolitsea*‐*Actinodaphne* (*Neo* and *Act* complex) and the trinerved *Lindera* complex (*Lindera* II) (ML analysis; Figure 5). However, according to the BI analysis, *Lindera* IV is grouped with *Parasassafras* first and then shares a sister relationship with other clades (Appendix S3). When only protein‐coding genes are used, the BI tree is similar in topology to the plastome‐based ML tree (Appendix S3). However, the ML analysis with the coding genes predicts a sister relationship between the clade containing *Parasassafras* and other clades including the *Lindera* IV clade (Appendix S3). A sister relationship between clade *Lindera* V and VI was strongly supported by all phylogenetic trees, while the phylogenetic location of these two clades was inconsistent according to analyses with the whole genome sequences and protein‐coding sequences. Based on the whole chloroplast genome analysis, they formed a larger clade with *Litsea* I, *Lindera* I to IV, *Parasassafras*, and the *Neo and Act complex* (Figure 5; Appendix S3), but are closer to the clade containing genus *Litsea* (*Litsea* II and III), *L. praecox* (Siebold and Zucc.) Blume from sect. *Lindera* and *L. rubronervia* Gamble from sect. *Sphaerocarpae* (*Lindera* VII), genus *Laurus* L. when using protein‐coding genes (Appendix S3).

The results all support a close relationship between *L. obtusiloba* (*Lindera* I) and the genus *Litsea* (*Litsea* I), and that the individuals have diverged into the Northern and the Southern lineages (Figure 5). However, analyses with both datasets failed to produce a species boundary between *L. obtusiloba* var. *obtusiloba* and *L. obtusiloba* var. *heterophylla*. Although the Northern population and the Southern population are separated in the phylogenetic trees, the *L. obtusiloba* individuals in each region are still clustered in a mixed way on the obtained tees.

### 3.4 Selection analyses

**FIGURE 6:**
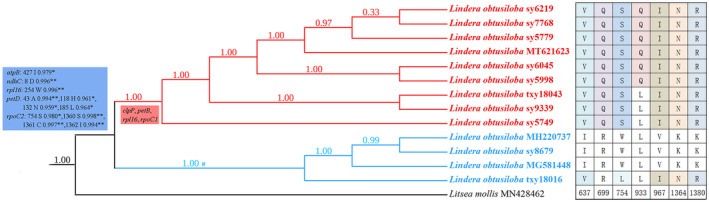
Phylogenetic tree reconstruction and positive selection genes of *Lindera obtusiloba* using the maximum likelihood (ML) method based on 79 coding sequences. Genes were colored with blue background were identified by site model, Genes were colored with red background were identified by branch model with the Northern population of *L. obtusiloba* as the foreground branch. Amino acid differences were observed between the Northern and the Southern populations of *L. obtusiloba* for *rpoC2* gene. *means *p* < 0.05 according selection analyses. **means *p* <0.01 according selection analyses.

Most protein‐coding genes are under purifying selection indicated by their low dN/dS ratios in the analyses with both the branch model and the site model. Five genes (*atpB*, *ndhC*, *rpl16*, *petD*, *rpoC2*) were found to have evolved under positive selection in the *L. obtusiloba* clade (Figure 5) according to Site model analysis. It was found that four sites were potentially under positive selection in *petD* and *rpoC2* and that only a single site was detected for *atpB*, *ndhC*, and *rpl16* (*p* < .05) (Appendix S4). Seven amino acid differences were observed between the Northern and the Southern populations of *L. obtusiloba* for *rpoC2* (Figure 6). The Branch model analysis supports strong selective pressure on *clpP*, *petB*, *rpl16*, and *rpoC1*, which put the Northern individuals into the foreground branch and the Southern individuals into the background branch (Appendix S4). However, the evolutionary significance of these sites cannot be accurately evaluated at this point due to the lack of 3D structures and functional analysis of these proteins.

## 4 DISCUSSION

In this study, intraspecific variations of *L. obtusiloba* were examined by analyzing 13 plastomes, covering the Northern and Southern geographic types. Despite their similarities in overall structure, gene order, and GC contents, the plastomes do vary in size, with *L. obtusiloba* Shandong being the largest. The variations in size could be attributed to the expansion of the IR regions in this sample (Abdullah, Mehmood, et al., 2021; Abdullah, Shahzadi, et al., 2021; Zhang et al., 2020). The chloroplast genome of *L. obtusiloba* from Shandong has a 678‐nucleotide insertion in its IR regions, which explains why it is bigger than other individuals (Table 2). A longer LSC with multiple InDels in the Northern population might also account for the size variation. These results reflect that both expansions in the IRs and InDel events can lead to variations in the plastomes at the intraspecific level (Jiang et al., 2017; Lei et al., 2016; Muraguri et al., 2020). Although the Northern samples contain larger chloroplast genomes, they have fewer parsimony informative sites and InDels when compared to the Southern individuals (Table 2), indicating a shorter evolutionary history and relatively homogeneous topography in North of East Asia (Tian et al., 2020). This is also supported by the fact that the Northern individuals possess less diversity in the SSR types and the number of repeats than the Southern population (Tian et al., 2020), which contradicts the results of a previous study by Ye et al. (2017). This difference may be related to the number of individuals and DNA target loci selected. Due to the presence of intraspecific variations, more representative populations need to be included when investigating genome diversity and intraspecific boundaries of species with a broad range of distribution.

The mVISTA results suggest that the coding regions are highly conserved and that variations are more likely to appear in the LSC and SSC regions than in the IR regions. This is consistent with previous findings with other Lauraceae species and flowering plants (Li, Luo, et al., 2021; Li, Yang, et al., 2021; Liu, Chen, et al., 2021; Liu, Ma, et al., 2021; Song et al., 2020; Yang et al., 2022). A total of four intergenic variable regions were identified across all the analyzed chloroplast genomes. Among these, two highly variable regions were found in the Northern population, but no high variable regions were detected in individuals from the Southern populations (Figure 3). It is speculated that the difference in cpDNA divergence patterns between the Northern and the Southern populations might be shaped by their specific evolutionary history (Tian et al., 2020). For the Northern populations, most species have a recent population history, which was formed via postglacial migration, while Southern populations may have served as glacial refugia during Pleistocene climate fluctuations (Milne & Abbott, 2002; Qiu et al., 2011; Ye et al., 2017). Additionally, the highly variable regions discovered in this study contain more variable sites when compared with one previous study (Ye et al., 2017). Based on their study, the *psbA*–*trnH*, *trnL*–*trnF*, *trnS*–*trnG*, and *rpl16* chloroplast DNA markers were designed. The variable regions detected in this study can also be used as potential DNA markers for studying the evolutionary history of *L. obtusiloba* and resolving the taxonomic status of *L. obtusiloba* with global sampling.

Phylogenetic trees were reconstructed using the chloroplast genomes of *L. obtusiloba* from different geographic regions and 51 plastomes of the “core Laureae.” Our results support the monophyletic origin of the “core Laureae” and the monophyletic status of the clade *Litsea* I, *Lindera* II, *Lindera* IV, *Lindera* V, and *Lindera* VI, which are consistent with some previous work (Jo et al., 2019; Liu, Chen, et al., 2021; Liu, Ma, et al., 2021; Song et al., 2020; Tian et al., 2019; Xiao et al., 2020). Phylogenetic results of the clade *Litsea* III, *Lindera* V, and *Lindera* VI in our studies are in agreement with a previous phylogenetic work by Zhao et al. (2018), who defined the relationships among the species located in sub‐clade I, and sub‐clade II containing *Lindera* sect. *Aperula*. However, the species, *L. benzoin* (L.) Blume distributed in North America showed a close relationship with the *Lindera* sect. *Daphnidium*, genus *Neolitsea*, and *Actinodaphne* (Figure 5), which is inconsistent with Zhao et al. (2018). This inconsistency can be explained by the difference in the samples used in phylogenetic reconstruction. In this study, the species of *L. obtusiloba* were placed in one of the *Litsea* clades (*Litsea* I; Figure 5), which agrees with the previous studies (Fijridiyanto & Murakami, 2009; Li et al., 2004; Liu, Chen, et al., 2021; Liu, Ma, et al., 2021; Song et al., 2020). *L. obtusiloba* usually has trifid, pentafidentire, or sometimes entire leaf margins, and pentanerved or trinerved veins, which is significantly different from other *Lindera* plants (Li, Conran, et al., 2008; Li, Li, & Li, 2008; Tian et al., 2020; Ye et al., 2017). The differences in the shape, arrangement, and pubescence of leaves may have a prominent influence on the rates of photosynthesis and transpiration, which might have allowed some variety to become better adapted to its particular environment (Chitwood & Sinha, 2016; Ding et al., 2020). In our study, five genes were identified with positively selected sites. These genes include the ATP subunit gene (*atpB*), NAD(P)H dehydrogenase complex gene (*ndhC*), small subunits of ribosome gene (*rpl16*), cytochrome b/f complex subunit proteins (*petD*), and DNA‐dependent RNA polymerase gene (*rpoC2*). Additionally, *rpoC2* is one of the largest chloroplast genes, encoding the RNA polymerase β, which is essential for the transcription of several photosynthesis‐related genes (Cummings et al., 1994). This gene possesses four positively selected sites, and seven nonsynonymous substitutions were detected at seven other sites (Figure 6). *L. obtusiloba* is the northernmost species of the family Lauraceae, which is highly sensitive to aridity, and it has evolved adaptively to its surrounding environments, presumably through the regulation of photosynthesis‐related genes and the modification of the photosynthetic system. Based on the phylogeny, *L. obtusiloba* shares a close relationship with the genus *Litsea*. However, there is no obvious morphological evidence supporting their close relationship (Li, Conran, et al., 2008; Li, Li, & Li, 2008). As a result, further study at both the molecular and morphological levels with more intensive sampling will be necessary.

The species boundary of *L. obtusiloba* between var. *obtusiloba* and var. *heterophylla* is unresolved in the current work. As a result, we inferred that gene flow might still occur between these two varieties, which is supported by the presence of multiple refuges in the Hengduan Mountains (Ye et al., 2017). In the current work, four photosynthesis‐related genes that are positively selected were identified in the Southern population, which suggests that environmental heterogeneity might have led to more intraspecific variations in this particular population (Tian et al., 2020). Together, these make it extremely challenging to distinguish the two varieties in the Southwest region of East Asia. Consequently, further examination potentially involving nuclear genomic data will be critical to fully elucidate the possibility of having hybridization or gene flow occur in the individuals from the admixture regions.

## 5 CONCLUSIONS

In this study, we investigated the intraspecific diversity of *L. obtusiloba* by comparative plastomic and phylogenetic analyses. The structural characteristics, gene content, nucleotide variations, InDel events, and sequence repeats of the 13 *L. obtusiloba* chloroplast genomes are highly conserved. Here, two groups congruent with the geographical distribution were found, and the aridity belt is responsible for this intraspecific divergence. Differences in nucleotide variable sites, simple sequence repeats, and variable hotspot regions support the discontinuous distribution of *L. obtusiloba* in Northern and Southern East Asia. *L. obtusiloba* in different regions has experienced different evolutionary pressures posed by various geographical, environmental, historical, and climatic factors, which have evolved a few photosynthesis‐related genes adaptively under positive selection. The phylogenetic confirmed the position of *L. obtusiloba* within the “core Laureae” and suggests that the relationship between *L. obtusiloba*, and genus *Litsea* should be provided more evidence. However, considering its wide distribution, a larger number of chloroplast and nuclear genomes should be included in further analysis to fully understand the evolutionary history of *L. obtusiloba*.

## AUTHOR CONTRIBUTIONS

**Xiangyu Tian:** Conceptualization (lead); data curation (lead); formal analysis (lead); investigation (lead); methodology (lead); resources (lead); software (lead); validation (lead); visualization (lead); writing – original draft (lead); writing – review and editing (lead). **Jia Guo:** Data curation (equal); funding acquisition (equal); investigation (equal); validation (equal); writing – review and editing (equal). **Yu Song:** Conceptualization (equal); funding acquisition (equal); project administration (lead); resources (lead); supervision (equal); writing – review and editing (equal). **Qunfei Yu:** Data curation (equal); formal analysis (equal); resources (equal). **Cao Liu:** Data curation (equal); formal analysis (equal); methodology (equal); resources (equal); visualization (equal). **Zhixi Fu:** Funding acquisition (equal); project administration (equal); visualization (equal); writing – review and editing (equal). **Yuhua Shi:** Investigation (equal); resources (equal); validation (equal); writing – review and editing (equal). **Yizhen Shao:** Methodology (equal); resources (equal); software (equal); validation (equal). **Zhiliang Yuan:** Conceptualization (equal); funding acquisition (equal); project administration (equal); resources (equal); supervision (equal); writing – review and editing (equal).

## FUNDING INFORMATION

This work was partially supported by grants from the Applied Basic Research Project of Yunnan (2019FB057), the National Natural Science Foundation of China (No. 32000158), and the Technologies Research and Development Program of Henan (No. 222102110009).

## CONFLICT OF INTEREST STATEMENT

The authors declare that they have no conflict of interest for the publication of the manuscript.

## Supporting information


Appendix S1.

Appendix S2.

Appendix S3.

Appendix S4.

## Data Availability

The *Lindera obtusiloba* plastomes generated in this study are available in the NCBI GenBank repository with accession numbers ON520645 to ON520654. The Supplementary data to this article can be found online at https://10.1002/ece3.11119.

## References

[ece311119-bib-0001] Abdullah, F. M. , Shahzadi, I. , Ali, Z. , Islam, M. , Naeem, M. , Mirza, B. , Lockhart, P. J. , Ahmed, I. , & Waheed, M. T. (2021). Correlations among oligonucleotide repeats, nucleotide substitutions, and insertion–deletion mutations in chloroplast genomes of plant family Malvaceae. Journal of Systematics and Evolution, 59(2), 388–402.

[ece311119-bib-0002] Abdullah, M. , Mehmood, F. , Heidari, P. , Rahim, A. , Ahmed, I. , & Poczai, P. (2021). Pseudogenization of the chloroplast threonine (*trnT‐GGU*) gene in the sunflower family (Asteraceae). Scientific Reports, 11(1), 21122. 34702873 10.1038/s41598-021-00510-4 PMC8548347

[ece311119-bib-0003] Alix, K. , Gérard, P. R. , Schwarzacher, T. , & Heslop‐Harrison, J. S. P. (2017). Polyploidy and interspecific hybridization: Partners for adaptation, speciation and evolution in plants. Annals of Botany, 120(2), 183–194. 28854567 10.1093/aob/mcx079 PMC5737848

[ece311119-bib-0004] Alqahtani, A. A. , & Jansen, R. K. (2021). The evolutionary fate of *rpl32* and *rps16* losses in the *Euphorbia schimperi* (Euphorbiaceae) plastome. Scientific Reports, 11(1), 7466. 33811236 10.1038/s41598-021-86820-z PMC8018952

[ece311119-bib-0005] Aschehoug, E. T. , Brooker, R. , Atwater, D. Z. , Maron, J. L. , & Callaway, R. M. (2016). The mechanisms and consequences of interspecific competition among plants. Annual Review of Ecology, Evolution, and Systematics, 47(1), 263–281.

[ece311119-bib-0006] Bai, W. N. , Wang, W. T. , & Zhang, D. Y. (2016). Phylogeographic breaks within Asian butternuts indicate the existence of a phytogeographic divide in East Asia. New Phytologist, 209(4), 1757–1772. 26499508 10.1111/nph.13711

[ece311119-bib-0007] Beier, S. , Thiel, T. , Münch, T. , Scholz, U. , & Mascher, M. (2017). MISA‐web: A web server for microsatellite prediction. Bioinformatics, 33(16), 2583–2585. 28398459 10.1093/bioinformatics/btx198 PMC5870701

[ece311119-bib-0008] Blomqvist, D. , Pauliny, A. , Larsson, M. , & Flodin, L. Å. (2010). Trapped in the extinction vortex? Strong genetic effects in a declining vertebrate population. BMC Evolutionary Biology, 10(1), 33. 20122269 10.1186/1471-2148-10-33 PMC2824661

[ece311119-bib-0009] Bürger, R. , Schneider, K. A. , & Willensdorfer, M. (2006). The conditions for speciation through intraspecific competition. Evolution, 60(11), 2185–2206. 17236413

[ece311119-bib-0010] Cao, Y. , Xuan, B. , Peng, B. , Li, C. , Chai, X. , & Tu, P. (2016). The genus *Lindera*: A source of structurally diverse molecules having pharmacological significance. Phytochemistry Reviews, 15(5), 869–906.

[ece311119-bib-0011] Chanderbali, A. , Van der Werff, H. , & Renner, S. (2001). Phylogeny and historical biogeography of Lauraceae: Evidence from the chloroplast and nuclear genomes. Annals of the Missouri Botanical Garden, 88(1), 104–134.

[ece311119-bib-0012] Chen, T. Y. , & Lou, A. R. (2019). Phylogeography and paleodistribution models of a widespread birch (*Betula platyphylla* Suk.) across East Asia: Multiple refugia, multidirectional expansion, and heterogeneous genetic pattern. Ecology and Evolution, 9, 7792–7807. 31346441 10.1002/ece3.5365 PMC6635942

[ece311119-bib-0013] Chitwood, D. H. , & Sinha, N. R. (2016). Evolutionary and environmental forces sculpting leaf development. Current Biology, 26(7), R297–R306. 27046820 10.1016/j.cub.2016.02.033

[ece311119-bib-0014] Cummings, M. P. , King, L. M. , & Kellogg, E. A. (1994). Slipped‐strand mispairing in a plastid gene: *rpoC2* in grasses (Poaceae). Molecular Biology and Evolution, 11(1), 1–8. 8121278 10.1093/oxfordjournals.molbev.a040084

[ece311119-bib-0015] Des Roches, S. , Post, D. M. , Turley, N. E. , Bailey, J. K. , Hendry, A. P. , Kinnison, M. T. , Schweitzer, J. A. , & Palkovacs, E. P. (2018). The ecological importance of intraspecific variation. Nature Ecology & Evolution, 2(1), 57–64. 29203921 10.1038/s41559-017-0402-5

[ece311119-bib-0016] Díaz, S. , Pascual, U. , Stenseke, M. , Martín‐López, B. , Watson, R. T. , Molnár, Z. , Hill, R. , Chan, K. M. A. , Baste, I. A. , Brauman, K. A. , Polasky, S. , Church, A. , Lonsdale, M. , Larigauderie, A. , Leadley, P. W. , van Oudenhoven, A. P. E. , van der Plaat, F. , Schröter, M. , Lavorel, S. , … Shirayama, Y. (2018). Assessing nature's contributions to people. Science, 359(6373), 270–272. 29348221 10.1126/science.aap8826

[ece311119-bib-0017] Ding, J. Y. , Johnson, E. A. , & Martin, Y. E. (2020). Optimization of leaf morphology in relation to leaf water status: A theory. Ecology and Evolution, 10(3), 1510–1525. 32076530 10.1002/ece3.6004 PMC7029057

[ece311119-bib-0018] Fijridiyanto, I. , & Murakami, N. (2009). Phylogeny of *Litsea* and related genera (Laureae‐Lauraceae) based on analysis of *rpb2* gene sequences. Journal of Plant Research, 122(3), 283–298. 19219578 10.1007/s10265-009-0218-8

[ece311119-bib-0019] Frazer, K. A. , Pachter, L. , Poliakov, A. , Rubin, E. M. , & Dubchak, I. (2004). VISTA: Computational tools for comparative genomics. Nucleic Acids Research, 32, W273–W279. 15215394 10.1093/nar/gkh458 PMC441596

[ece311119-bib-0020] Freise, C. , Erben, U. , Neuman, U. , Kim, K. , Zeitz, M. , Somasundaram, R. , & Ruehl, M. (2010). An active extract of *Lindera obtusiloba* inhibits adipogenesis via sustained Wnt signaling and exerts anti‐inflammatory effects in the 3T3‐L1 preadipocytes. The Journal of Nutritional Biochemistry, 21(12), 1170–1177. 20092995 10.1016/j.jnutbio.2009.09.013

[ece311119-bib-0021] Gao, F. , Chen, C. , Arab, D. A. , du, Z. , He, Y. , & Ho, S. Y. W. (2019). EasyCodeML: A visual tool for analysis of selection using CodeML. Ecology and Evolution, 9(7), 3891–3898. 31015974 10.1002/ece3.5015 PMC6467853

[ece311119-bib-0022] Gray, L. N. , Barley, A. J. , Poe, S. , Thomson, R. C. , Nieto‐Montes de Oca, A. , & Wang, I. J. (2019). Phylogeography of a widespread lizard complex reflects patterns of both geographic and ecological isolation. Molecular Ecology, 28(3), 644–657. 30525264 10.1111/mec.14970

[ece311119-bib-0024] Guo, Z. , Sun, B. , Zhang, Z. , Peng, S. , Xiao, G. , Ge, J. , Hao, Q. , Qiao, Y. , Liang, M. , Liu, J. , Yin, Q. , & Wei, J. J. (2008). A major reorganization of Asian climate by the early Miocene. Climate of the Past, 4(3), 153–174.

[ece311119-bib-0025] Halbritter, A. H. , Fior, S. , Keller, I. , Billeter, R. , Edwards, P. J. , Holderegger, R. , Karrenberg, S. , Pluess, A. R. , Widmer, A. , & Alexander, J. M. (2018). Trait differentiation and adaptation of plants along elevation gradients. Journal of Evolutionary Biology, 31(6), 784–800. 29518274 10.1111/jeb.13262

[ece311119-bib-0026] Hall, T. , Biosciences, I. , & Carlsbad, C. (2011). BioEdit: An important software for molecular biology. GERF Bulletin of Biosciences, 2(1), 60–61.

[ece311119-bib-0027] Hong, C. O. , Rhee, C. H. , Won, N. H. , Choi, H. D. , & Lee, K. W. (2013). Protective effect of 70% ethanolic extract of *Lindera obtusiloba* Blume on tert‐butyl hydroperoxide‐induced oxidative hepatotoxicity in rats. Food and Chemical Toxicology, 53, 214–220. 23211441 10.1016/j.fct.2012.11.032

[ece311119-bib-0028] Huang, C. L. , Chen, J. H. , Chang, C. T. , Chung, J. D. , Liao, P. C. , Wang, J. C. , & Hwang, S. Y. (2016). Disentangling the effects of isolation‐by‐distance and isolation‐by‐environment on genetic differentiation among *rhododendron* lineages in the subgenus Tsutsusi. Tree Genetics & Genomes, 12(3), 53.

[ece311119-bib-0029] Jiang, D. , Zhao, Z. , Zhang, T. , Zhong, W. , Liu, C. , Yuan, Q. , & Huang, L. (2017). The chloroplast genome sequence of *Scutellaria baicalensis* provides insight into intraspecific and interspecific chloroplast genome diversity in Scutellaria. Genes, 8(9), 227. 28902130 10.3390/genes8090227 PMC5615360

[ece311119-bib-0030] Jin, J. J. , Yu, W. B. , Yang, J. B. , Song, Y. , de Pamphilis, C. W. , Yi, T. S. , & Li, D. Z. (2020). GetOrganelle: A fast and versatile toolkit for accurate de novo assembly of organelle genomes. Genome Biology, 21(1), 241. 32912315 10.1186/s13059-020-02154-5 PMC7488116

[ece311119-bib-0031] Jo, S. , Kim, Y. K. , Cheon, S. H. , Fan, Q. , & Kim, K. J. (2019). Characterization of 20 complete plastomes from the tribe Laureae (Lauraceae) and distribution of small inversions. PLoS One, 14(11), e0224622. 31675370 10.1371/journal.pone.0224622 PMC6824564

[ece311119-bib-0032] Kalyaanamoorthy, S. , Minh, B. Q. , Wong, T. K. F. , von Haeseler, A. , & Jermiin, L. S. (2017). ModelFinder: Fast model selection for accurate phylogenetic estimates. Nature Methods, 14(6), 587–589. 28481363 10.1038/nmeth.4285 PMC5453245

[ece311119-bib-0033] Katoh, K. , & Standley, D. M. (2013). MAFFT multiple sequence alignment software version 7: Improvements in performance and usability. Molecular Biology and Evolution, 30(4), 772–780. 23329690 10.1093/molbev/mst010 PMC3603318

[ece311119-bib-0034] Kurtz, S. , Choudhuri, J. V. , Ohlebusch, E. , Schleiermacher, C. , Stoye, J. , & Giegerich, R. (2001). REPuter: The manifold applications of repeat analysis on a genomic scale. Nucleic Acids Research, 29(22), 4633–4642. 11713313 10.1093/nar/29.22.4633 PMC92531

[ece311119-bib-0035] Lee, C. , Choi, I.‐S. , Cardoso, D. , de Lima, H. C. , de Queiroz, L. P. , Wojciechowski, M. F. , Jansen, R. K. , & Ruhlman, T. A. (2021). The chicken or the egg? Plastome evolution and an independent loss of the inverted repeat in papilionoid legumes. The Plant Journal, 107(3), 861–875. 34021942 10.1111/tpj.15351

[ece311119-bib-0036] Lehwark, P. , & Greiner, S. (2019). GB2sequin ‐ a file converter preparing custom GenBank files for database submission. Genomics, 111(4), 759–761. 29842948 10.1016/j.ygeno.2018.05.003

[ece311119-bib-0037] Lei, W. , Ni, D. , Wang, Y. , Shao, J. , Wang, X. , Yang, D. , Wang, J. , Chen, H. , & Liu, C. (2016). Intraspecific and heteroplasmic variations, gene losses and inversions in the chloroplast genome of Astragalus membranaceus. Scientific Reports, 6(1), 21669. 26899134 10.1038/srep21669 PMC4761949

[ece311119-bib-0038] Leigh, D. M. , Hendry, A. P. , Vázquez‐Domínguez, E. , & Friesen, V. L. (2019). Estimated six per cent loss of genetic variation in wild populations since the industrial revolution. Evolutionary Applications, 12(8), 1505–1512. 31462910 10.1111/eva.12810 PMC6708419

[ece311119-bib-0039] Li, H. T. , Luo, Y. , Gan, L. , Ma, P. F. , Gao, L. M. , Yang, J. B. , Cai, J. , Gitzendanner, M. A. , Fritsch, P. W. , Zhang, T. , Jin, J. J. , Zeng, C. X. , Wang, H. , Yu, W. B. , Zhang, R. , van der Bank, M. , Olmstead, R. G. , Hollingsworth, P. M. , Chase, M. W. , … Li, D. Z. (2021). Plastid phylogenomic insights into relationships of all flowering plant families. BMC Biology, 19(1), 232. 34711223 10.1186/s12915-021-01166-2 PMC8555322

[ece311119-bib-0040] Li, H. W. (1985). Parallel evolution in *Listea* and *Lindera* of Lauraceae. Acta Botanica Yunnanica, 7(2), 129–135.

[ece311119-bib-0041] Li, J. , Christophel, D. , Conran, J. , & Li, H. W. (2004). Phylogenetic relationships within the ‘core’ Laureae (*Litsea* complex, Lauraceae) inferred from sequences of the chloroplast gene *matK* and nuclear ribosomal DNA ITS regions. Plant Systematics and Evolution, 246(1–2), 19–34.

[ece311119-bib-0042] Li, J. , Conran, J. G. , Christophel, D. C. , Li, Z. M. , Li, L. , & Li, H. W. (2008). Phylogenetic relationships of the *Litsea* Complex and Core Laureae (Lauraceae) using ITS and ETS sequences and morphology. Annals of the Missouri Botanical Garden, 95, 580–599.

[ece311119-bib-0043] Li, L. , Li, J. , Conran, J. G. , Li, X. W. , & Li, H. W. (2007). Phylogeny of *Neolitsea* (Lauraceae) inferred from Bayesian analysis of nrDNA ITS and ETS sequences. Plant Systematics and Evolution, 269(3–4), 203–221.

[ece311119-bib-0044] Li, S. G. , Li, X. W. , & Li, J. (2008). Lauraceae. In Z. Y. Wu , P. H. Raven , & D. Y. Hong (Eds.), Flora of China (Vol. 7, pp. 102–254). Science Press, Beijing & Missouri Botanical Garden Press.

[ece311119-bib-0045] Li, X. , Yang, J. B. , Wang, H. , Song, Y. , Corlett, R. T. , Yao, X. , Li, D. Z. , & Yu, W. B. (2021). Plastid NDH Pseudogenization and gene loss in a recently derived lineage from the largest Hemiparasitic plant genus *Pedicularis* (Orobanchaceae). Plant and Cell Physiology, 62(6), 971–984. 34046678 10.1093/pcp/pcab074 PMC8504446

[ece311119-bib-0046] Liu, C. , Chen, H. , Tang, L. , Khine, P. K. , Han, L. H. , Song, Y. , & Tan, Y. H. (2021). Plastid genome evolution of a monophyletic group in the subtribe Lauriineae (Laureae, Lauraceae). Plant Diversity, 44(4), 377–388. 35967258 10.1016/j.pld.2021.11.009 PMC9363652

[ece311119-bib-0047] Liu, Z. F. , Ma, H. , Ci, X. Q. , Li, L. , Song, Y. , Liu, B. , Li, H. W. , Wang, S. L. , Qu, X. J. , Hu, J. L. , Zhang, X. Y. , Conran, J. G. , Twyford, A. D. , Yang, J. B. , Hollingsworth, P. M. , & Li, J. (2021). Can plastid genome sequencing be used for species identification in Lauraceae? Botanical Journal of the Linnean Society, 197(1), 1–14.

[ece311119-bib-0048] Maple, J. , & Møller, S. G. (2006). Plastid division: Evolution, mechanism and complexity. Annals of Botany, 99(4), 565–579. 17138581 10.1093/aob/mcl249 PMC2802928

[ece311119-bib-0049] Martín‐Serra, A. , & Benson, R. B. J. (2020). Developmental constraints do not influence long‐term phenotypic evolution of marsupial forelimbs as revealed by interspecific disparity and integration patterns. The American Naturalist, 195(3), 547–560. 10.1086/707194 32097034

[ece311119-bib-0050] Milne, R. I. , & Abbott, R. J. (2002). The origin and evolution of tertiary relict floras. Advances in Botanical Research, 38, 281–314.

[ece311119-bib-0051] Mimura, M. , Yahara, T. , Faith, D. P. , Vázquez‐Domínguez, E. , Colautti, R. I. , Araki, H. , Javadi, F. , Núñez‐Farfán, J. , Mori, A. S. , Zhou, S. , Hollingsworth, P. M. , Neaves, L. E. , Fukano, Y. , Smith, G. F. , Sato, Y. I. , Tachida, H. , & Hendry, A. P. (2017). Understanding and monitoring the consequences of human impacts on intraspecific variation. Evolutionary Applications, 10(2), 121–139. 28127389 10.1111/eva.12436 PMC5253428

[ece311119-bib-0052] Muraguri, S. , Xu, W. , Chapman, M. , Muchugi, A. , Oluwaniyi, A. , Oyebanji, O. , & Liu, A. (2020). Intraspecific variation within Castor bean (*Ricinus communis* L.) based on chloroplast genomes. Industrial Crops and Products, 155, 112779.

[ece311119-bib-0053] Nguyen, L. T. , Schmidt, H. A. , von Haeseler, A. , & Minh, B. Q. (2014). IQ‐TREE: A fast and effective stochastic algorithm for estimating maximum‐likelihood phylogenies. Molecular Biology and Evolution, 32(1), 268–274. 25371430 10.1093/molbev/msu300 PMC4271533

[ece311119-bib-0054] Qiu, Y. X. , Fu, C. X. , & Comes, H. P. (2011). Plant molecular phylogeography in China and adjacent regions: Tracing the genetic imprints of quaternary climate and environmental change in the world's most diverse temperate flora. Molecular Phylogenetics and Evolution, 59(1), 225–244. 21292014 10.1016/j.ympev.2011.01.012

[ece311119-bib-0055] Qu, X. J. , Moore, M. J. , Li, D. Z. , & Yi, T. S. (2019). PGA: A software package for rapid, accurate, and flexible batch annotation of plastomes. Plant Methods, 15(1), 50. 31139240 10.1186/s13007-019-0435-7 PMC6528300

[ece311119-bib-0056] Rohwer, J. G. (2000). Toward a phylogenetic classification of the Lauraceae: Evidence from *matK* sequences. Systematic Botany, 25, 60–71.

[ece311119-bib-0057] Ronquist, F. , Teslenko, M. , van der Mark, P. , Ayres, D. L. , Darling, A. , Höhna, S. , Larget, B. , Liu, L. , Suchard, M. A. , & Huelsenbeck, J. P. (2012). MrBayes 3.2: Efficient Bayesian phylogenetic inference and model choice across a large model space. Systematic Biology, 61(3), 539–542. 22357727 10.1093/sysbio/sys029 PMC3329765

[ece311119-bib-0058] Rozas, J. , Ferrer‐Mata, A. , Sánchez‐DelBarrio, J. C. , Guirao‐Rico, S. , Librado, P. , Ramos‐Onsins, S. E. , & Sánchez‐Gracia, A. (2017). DnaSP 6: DNA sequence polymorphism analysis of large data sets. Molecular Biology and Evolution, 34(12), 3299–3302. 29029172 10.1093/molbev/msx248

[ece311119-bib-0059] Ruckelshaus, M. H. , Jackson, S. T. , Mooney, H. A. , Jacobs, K. L. , Kassam, K. A. S. , Arroyo, M. T. K. , Báldi, A. , Bartuska, A. M. , Boyd, J. , Joppa, L. N. , Kovács‐Hostyánszki, A. , Parsons, J. P. , Scholes, R. J. , Shogren, J. F. , & Ouyang, Z. (2020). The IPBES global assessment: Pathways to action. Trends in Ecology & Evolution, 35(5), 407–414. 32294422 10.1016/j.tree.2020.01.009

[ece311119-bib-0060] Ruhlman, T. A. , & Jansen, R. K. (2014). The plastid genomes of flowering plants. In Chloroplast biotechnology (pp. 3–38). Springer. 10.1007/978-1-62703-995-6_1 24599844

[ece311119-bib-0061] Sibbald, S. J. , & Archibald, J. M. (2020). Genomic insights into plastid evolution. Genome Biology and Evolution, 12(7), 978–990. 32402068 10.1093/gbe/evaa096 PMC7348690

[ece311119-bib-0062] Song, Y. , Yu, W.‐B. , Tan, Y.‐H. , Jin, J.‐J. , Wang, B. , Yang, J.‐B. , Liu, B. , & Corlett, R. T. (2020). Plastid Phylogenomics improve phylogenetic resolution in the Lauraceae. Journal of Systematics and Evolution, 58(4), 423–439.

[ece311119-bib-0063] Taudt, A. , Colomé‐Tatché, M. , & Johannes, F. (2016). Genetic sources of population epigenomic variation. Nature Reviews Genetics, 17(6), 319–332. 10.1038/nrg.2016.45 27156976

[ece311119-bib-0064] Tian, X. Y. , Ye, J. W. , & Song, Y. (2019). Plastome sequences help to improve the systematic position of Trinerved *Lindera* species in the family Lauraceae. PeerJ, 7, e7662. 31608166 10.7717/peerj.7662 PMC6786250

[ece311119-bib-0065] Tian, X. Y. , Ye, J. W. , Wang, T. M. , Bao, L. , & Wang, H. F. (2020). Different processes shape the patterns of divergence in the nuclear and chloroplast genomes of a relict tree species in East Asia. Ecology and Evolution, 10(10), 4331–4342. 32489600 10.1002/ece3.6200 PMC7246201

[ece311119-bib-0066] Tillich, M. , Lehwark, P. , Pellizzer, T. , Ulbricht‐Jones, E. S. , Fischer, A. , Bock, R. , & Greiner, S. (2017). GeSeq – Versatile and accurate annotation of organelle genomes. Nucleic Acids Research, 45(W1), W6–W11. 28486635 10.1093/nar/gkx391 PMC5570176

[ece311119-bib-0067] Tonti‐Filippini, J. , Nevill, P. G. , Dixon, K. , & Small, I. (2017). What can we do with 1000 plastid genomes? The Plant Journal, 90(4), 808–818. 28112435 10.1111/tpj.13491

[ece311119-bib-0068] Tsui, H. P. (1987). A study on the system of *Lindera* . Acta Phytotaxonomica Sinica, 25(3), 161–171.

[ece311119-bib-0069] Wilting, A. , Courtiol, A. , Christiansen, P. , Niedballa, J. , Scharf, A. K. , Orlando, L. , Balkenhol, N. , Hofer, H. , Kramer‐Schadt, S. , Fickel, J. , & Kitchener, A. C. (2015). Planning tiger recovery: Understanding intraspecific variation for effective conservation. Science Advances, 1(5), e1400175. 26601191 10.1126/sciadv.1400175 PMC4640610

[ece311119-bib-0070] Xiao, T. W. , Xu, Y. , Jin, L. , Liu, T. J. , Yan, H. F. , & Ge, X. J. (2020). Conflicting phylogenetic signals in plastomes of the tribe Laureae (Lauraceae). PeerJ, 8, e10155. 33088627 10.7717/peerj.10155 PMC7568859

[ece311119-bib-0071] Xiao, T. W. , Yan, H. F. , & Ge, X. J. (2022). Plastid phylogenomics of tribe Perseeae (Lauraceae) yields insights into the evolution of east Asian subtropical evergreen broad‐leaved forests. BMC Plant Biology, 22(1), 32. 35027008 10.1186/s12870-021-03413-8 PMC8756638

[ece311119-bib-0072] Xu, J. H. , Liu, Q. , Hu, W. , Wang, T. , Xue, Q. , & Messing, J. (2015). Dynamics of chloroplast genomes in green plants. Genomics, 106(4), 221–231. 26206079 10.1016/j.ygeno.2015.07.004

[ece311119-bib-0073] Xu, L. L. , Yu, R. M. , Lin, X. R. , Zhang, B. W. , Li, N. , Lin, K. , Zhang, D. Y. , & Bai, W. N. (2021). Different rates of pollen and seed gene flow cause branch‐length and geographic cytonuclear discordance within Asian butternuts. New Phytologist, 232(1), 388–403. 34143496 10.1111/nph.17564 PMC8519134

[ece311119-bib-0074] Yang, T. , Sahu, S. K. , Yang, L. , Liu, Y. , Mu, W. , Liu, X. , Strube, M. L. , Liu, H. , & Zhong, B. (2022). Comparative analyses of 3,654 plastid genomes unravel insights into evolutionary dynamics and phylogenetic discordance of green plants. Frontiers in Plant Science, 13, 808156. 35498716 10.3389/fpls.2022.808156 PMC9038950

[ece311119-bib-0075] Yang, Z. (1998). Likelihood ratio tests for detecting positive selection and application to primate lysozyme evolution. Molecular Biology and Evolution, 15(5), 568–573. 9580986 10.1093/oxfordjournals.molbev.a025957

[ece311119-bib-0076] Ye, J. W. , Bai, W. N. , Bao, L. , Wang, T. M. , Wang, H. F. , & Ge, J. P. (2017). Sharp genetic discontinuity in the aridity‐sensitive *Lindera obtusiloba* (Lauraceae): Solid evidence supporting the tertiary floral subdivision in East Asia. Journal of Biogeography, 44(9), 2082–2095.

[ece311119-bib-0077] Zekun, L. , & Haixia, C. (2012). GC‐MS analysis of essential oil from the bark of *Lindera obtusiloba* . Chemistry of Natural Compounds, 48(4), 696–697.

[ece311119-bib-0078] Zhang, R. S. , Yang, J. , Hu, H. L. , Xia, R. X. , Li, Y. P. , Su, J. F. , Li, Q. , Liu, Y. Q. , & Qin, L. (2020). A high level of chloroplast genome sequence variability in the sawtooth oak *Quercus acutissima* . International Journal of Biological Macromolecules, 152, 340–348. 32109476 10.1016/j.ijbiomac.2020.02.201

[ece311119-bib-0079] Zhang, Y. H. , Wang, I. J. , Comes, H. P. , Peng, H. , & Qiu, Y. X. (2016). Contributions of historical and contemporary geographic and environmental factors to phylogeographic structure in a tertiary relict species, *Emmenopterys henryi* (Rubiaceae). Scientific Reports, 6, 24041. 27137438 10.1038/srep24041 PMC4853719

[ece311119-bib-0080] Zhang, Y. J. , Liang, T. T. , & Zang, D. K. (2014). Analysis on community composition and structure of *Lindera obtusiloba* in Laoshan. Journal of Agriculture, 4(4), 60–63.

[ece311119-bib-0081] Zhao, M. L. , Song, Y. , Ni, J. , Yao, X. , Tan, Y. H. , & Xu, Z. F. (2018). Comparative chloroplast genomics and phylogenetics of nine *Lindera* species (Lauraceae). Scientific Reports, 8(1), 8844. 29891996 10.1038/s41598-018-27090-0 PMC5995902

[ece311119-bib-0082] Zheng, S. , Poczai, P. , Hyvönen, J. , Tang, J. , & Amiryousefi, A. (2020). Chloroplot: An online program for the versatile plotting of organelle genomes. Frontiers in Genetics, 11, 576124. 33101394 10.3389/fgene.2020.576124 PMC7545089

