# Supplementary figures and images for: Intraspecific differentiation of Lindera obtusiloba as revealed by comparative plastomic and evolutionary analyses
Source: Ecol Evol. 2024 Mar 11;14(3):e11119. doi: 10.1002/ece3.11119 (PMC10927362; doi:10.1002/ece3.11119)

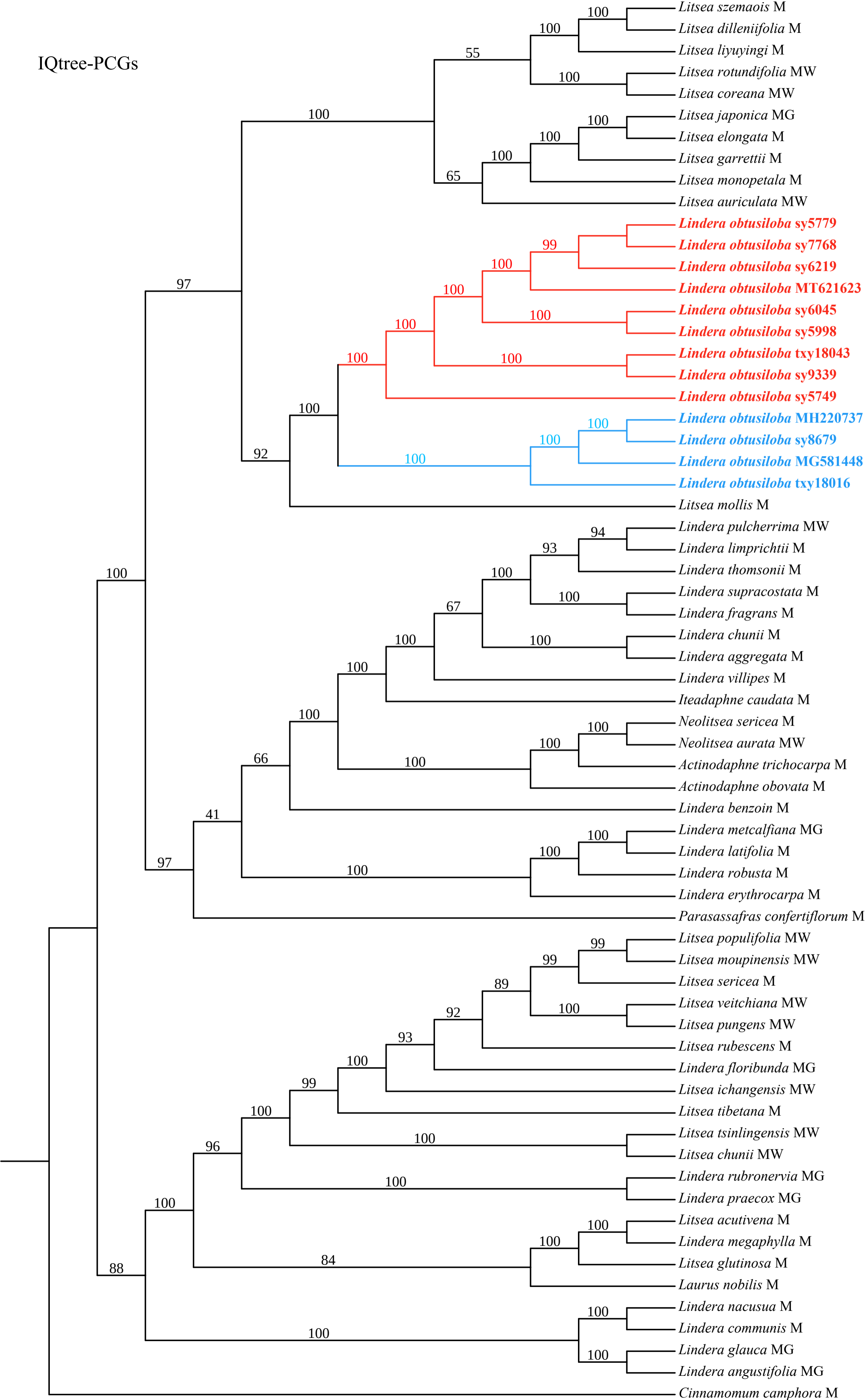

# MrBayes-PCGs

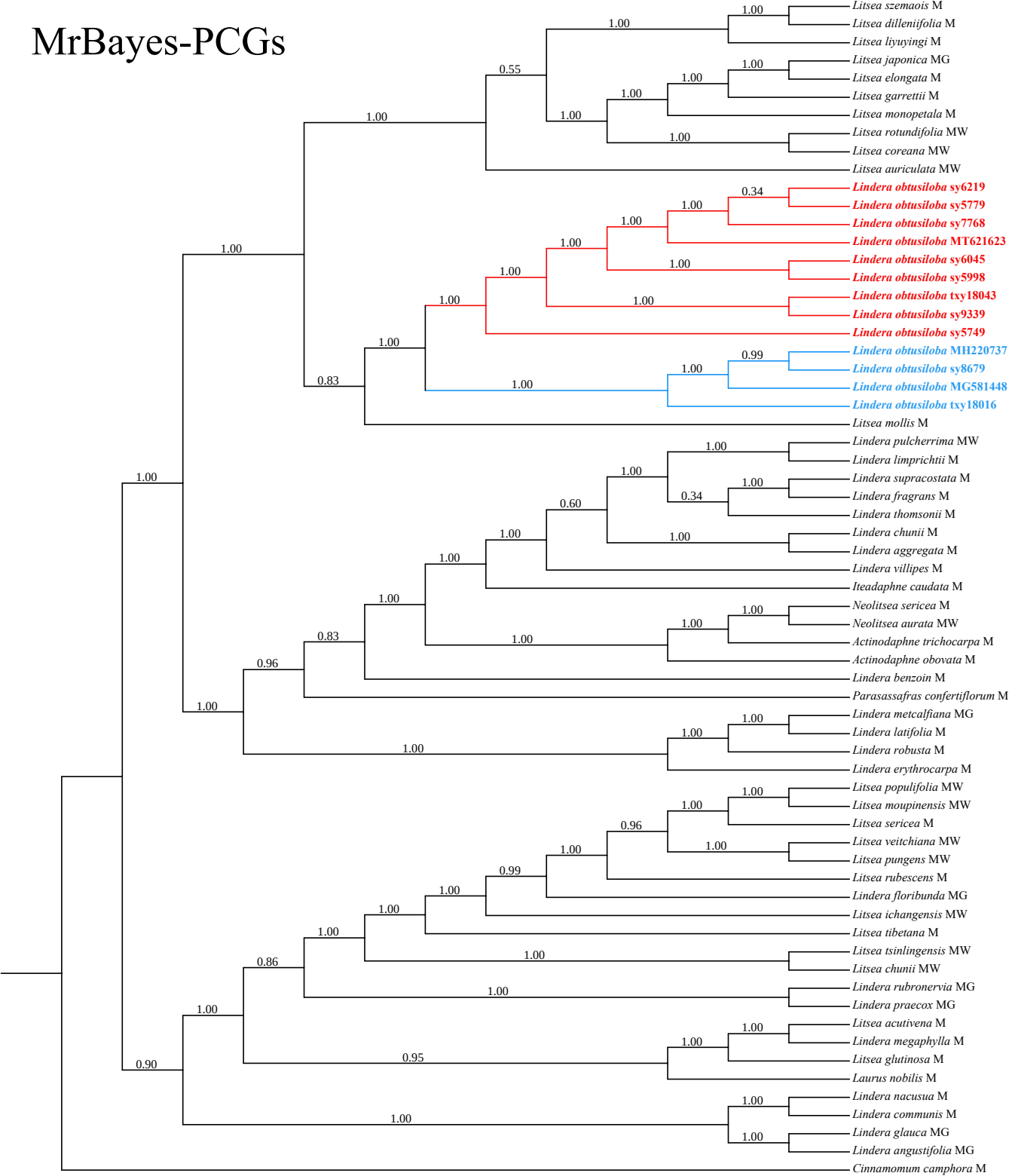

MrBayes-Whole chloroplast genomes

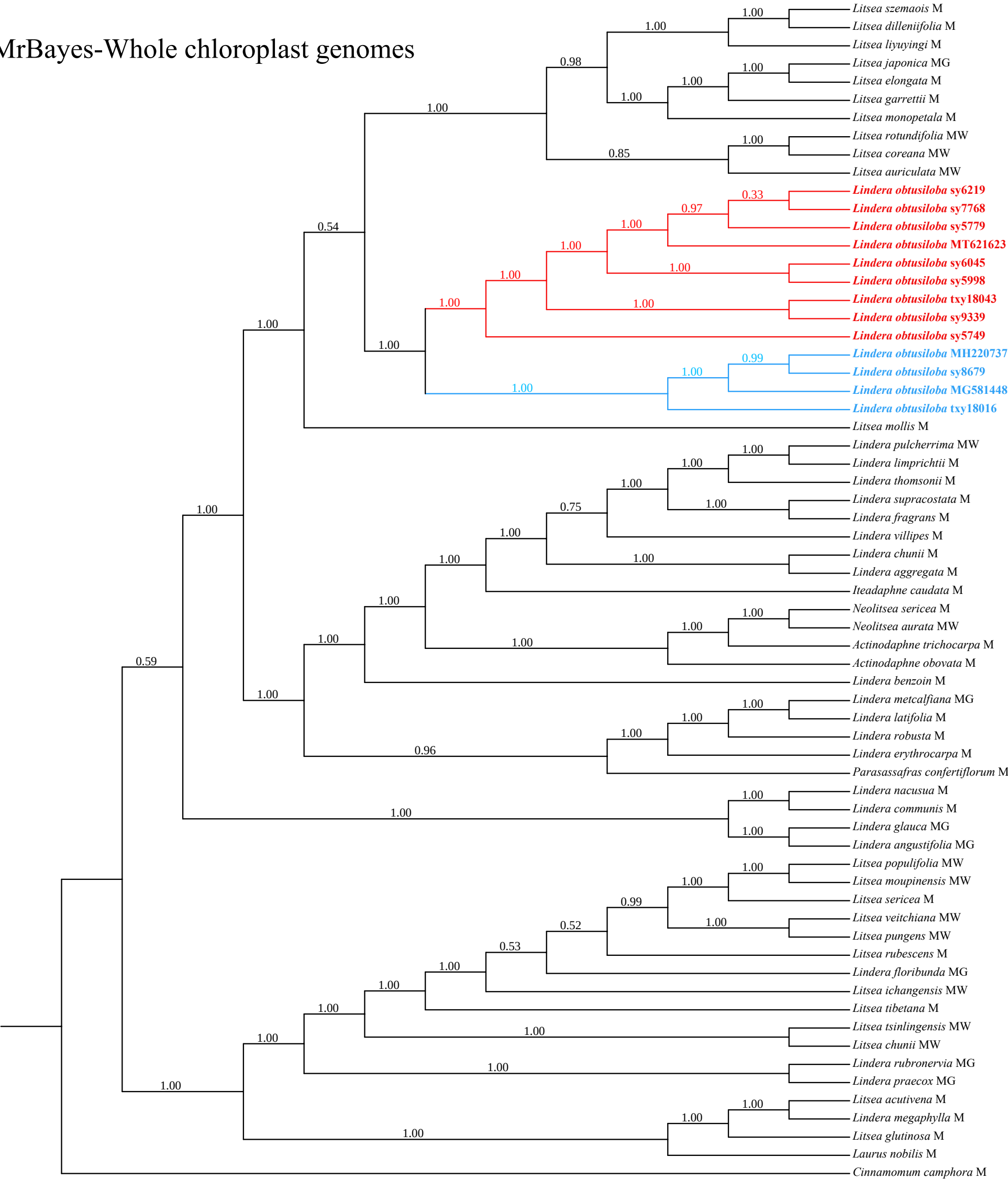

Supplement: Supplementary file 3 — Appendix S3. [file ECE3-14-e11119-s004.pdf]
